# Prodromic Inflammatory–Oxidative Stress in Peritoneal Leukocytes of Triple-Transgenic Mice for Alzheimer’s Disease

**DOI:** 10.3390/ijms25136976

**Published:** 2024-06-26

**Authors:** Noemí Ceprián, Irene Martínez de Toda, Ianire Maté, Antonio Garrido, Lydia Gimenez-Llort, Mónica De la Fuente

**Affiliations:** 1 Animal Physiology Unit, Department of Genetics, Physiology and Microbiology, Faculty of Biological Sciences, Complutense University of Madrid, 28040 Madrid, Spain; nceprian@ucm.es (N.C.); mondelaf@ucm.es (M.D.l.F.); 2 Institute of Investigation Hospital 12 Octubre (imas12), 28041 Madrid, Spain; 3 Department of Immunology, Microbiology and Parasitology, Faculty of Pharmacy, University of the Basque Country, 01006 Vitoria-Gasteiz, Spain; ianire.mate@ehu.eus; 4 Department of Biosciences, Faculty of Biomedical and Health Sciences, Universidad Europea de Madrid, 28670 Madrid, Spain; antonio.garrido@universidadeuropea.es; 5 Department of Psychiatry and Forensic Medicine, Institute of Neuroscience, School of Medicine, Universitat Autònoma de Barcelona, Cerdanyola del Vallès, 08193 Barcelona, Spain; lidia.gimenez@uab.cat

**Keywords:** cytokines, inflammation, longevity, oxidative damage, triple-transgenic mice for Alzheimer’s disease (3xTgAD)

## Abstract

Inflammatory–oxidative stress is known to be pivotal in the pathobiology of Alzheimer’s disease (AD), but the involvement of this stress at the peripheral level in the disease’s onset has been scarcely studied. This study investigated the pro-inflammatory profile and oxidative stress parameters in peritoneal leukocytes from female triple-transgenic mice for AD (3xTgAD) and non-transgenic mice (NTg). Peritoneal leukocytes were obtained at 2, 4, 6, 12, and 15 months of age. The concentrations of TNFα, INFγ, IL-1β, IL-2, IL-6, IL-17, and IL-10 released in cultures without stimuli and mitogen concanavalin A and lipopolysaccharide presence were measured. The concentrations of reduced glutathione (GSH), oxidized glutathione (GSSG), lipid peroxidation, and Hsp70 were also analyzed in the peritoneal cells. Our results showed that although there was a lower release of pro-inflammatory cytokines by 3xTgAD mice, this response was uncontrolled and overstimulated, especially at a prodromal stage at 2 months of age. In addition, there were lower concentrations of GSH in leukocytes from 3xTgAD and higher amounts of lipid peroxides at 2 and 4 months, as well as, at 6 months, a lower concentration of Hsp70. In conclusion, 3xTgAD mice show a worse pro-inflammatory response and higher oxidative stress than NTg mice during the prodromal stages, potentially supporting the idea that Alzheimer’s disease could be a consequence of peripheral alteration in the leukocyte inflammation–oxidation state.

## 1. Introduction

Alzheimer’s disease (AD) is the most common cause of dementia, and it is estimated that more than 130 million people will be affected by 2050 [1]. AD is a multifactorial disease caused by interactions of biological and environmental factors [2,3,4,5]. Due to its complexity, several hypotheses have been proposed to explain its pathobiology [4,6]. The main pathophysiologic manifestations of the disease are senile plaques, formed by the accumulation of β-amyloid, and neurofibrillary tangles, composed of hyperphosphorylation of the cytoskeletal protein tau. Both events lead to cell damage and microglial activation, inducing inflammation and oxidative stress [7]. In this context, inflammation and oxidative stress may be essential in its onset and progression [8,9,10,11].

While the precise moment when inflammation and oxidative stress manifest remains uncertain, it is widely recognized that disturbances in cellular homeostasis and the presence of β-amyloid can activate microglia, acting as damage-associated molecular patterns (DAMPs) [12,13]. Moreover, new studies suggest that β-amyloid may act as a cytokine [14]. Cytokines are essential in the communications between the immune and the nervous systems [15,16,17]. Even peripheral cytokines produced by tissue-resident leukocytes or peripheral blood leukocytes affect behavior and induce changes in the brain, altering the neuroendocrine function or neurotransmitter metabolism [16,18,19]. This fact is favored by increased blood–brain barrier permeability in AD [20]. Current studies in peripheral cytokines of patients with mild cognitive impairment (MCI) and AD have shown different cytokine concentrations between them and compared with healthy subjects [21,22,23]. On top of that, it has been proposed that infections may be crucial factors in developing the disease [19,24,25,26,27].

The brain is especially susceptible to oxidative damage due to its high metabolism, high glucose utilization, high polyunsaturated fatty acids, high presence of metabolites with oxidative capacity (i.e., redox-active transition metals, neurotransmitters), low antioxidant content, and limited capacity for regeneration [7,28]. It has been observed that mitochondrial alteration occurs even before the appearance of β-amyloid [29], which would be directly related to an alteration in the production of free radicals and, therefore, an increase in oxidative stress. Furthermore, oxidative and inflammatory stress are intimately linked and mutually reinforce each other [18]. For these reasons, oxidative and inflammatory stress are clinically relevant in searching for biomarkers and possible therapeutic targets [30,31,32,33,34,35].

Many animal models have been studied to understand the mechanism underlying the physiopathology of the disease and find possible biomarkers, especially in rodents [36,37,38]. These models, which are based on the familiar form of the disease, have improved the knowledge of the disease. The triple-transgenic mice model of AD (3xTgAD) is the one that better represents the neuropathological manifestations in an age-dependent and region-specific manner as that in the human brain. This model harbors tau_P310L_ and AβPP_swe_ transgenes in a knock-in mutant for PS1M146V, and it is the first and unique animal model that closely mimics both neuropathological manifestations: amyloid-β plaques and neurofibrillary tangles [39,40,41]. Previously, our group studied the changes with age in several functions and parameters of the redox state of peritoneal leukocytes in a longitudinal study of female 3xTgAD mice. These animals showed an impaired immune function and an increased redox state, particularly the latter, before the onset of the disease, which makes these parameters possible preclinical and prodromal markers of the disease [42]. Moreover, this mouse model has shown elevated levels of cytokines in plasma [43] and spleen [44] in late adult mice. Furthermore, they showed an exacerbated response to infections [45]. 

Because of this, the present work aimed to study longitudinally, before the onset (2 months of age) until the complete establishment (15 months) of the disease, several parameters of the inflammatory response and oxidative stress state of peritoneal leukocytes, as well as the longevity of female 3xTgAD and non-transgenic wild-type mice.

## 2. Results

### 2.1. Inflammatory Stress

**Figure 1:**
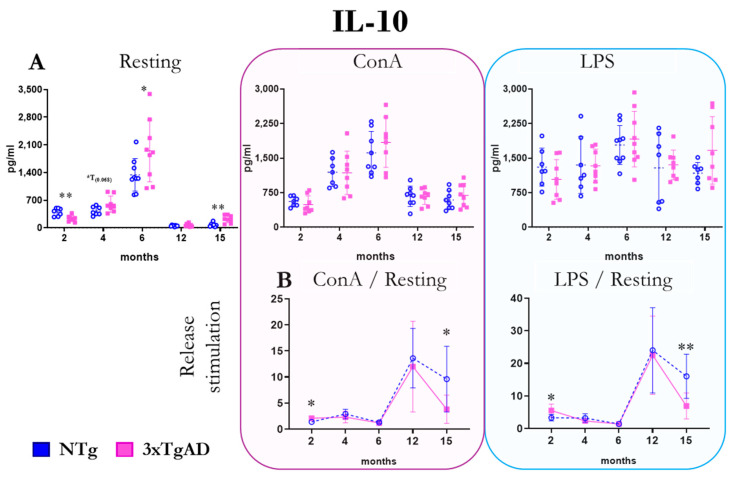
Changes with age in the release of the anti-inflammatory cytokine interleukin ten (IL-10) by peritoneal leukocytes from non-transgenic (NTg) and triple-transgenic mice for Alzheimer’s disease (3xTgAD). (**A**): In the absence of stimulation (resting condition) and in the presence of the mitogens concanavalin A (ConA) and lipopolysaccharide (LPS). (**B**): Stimulation release (relating values in the presence and absence of mitogenic stimulus). * *p* ≤ 0.05, ** *p* ≤ 0.01, differences between NTg and 3xTgAD.

To determine the differences in the inflammatory stress between non-transgenic (NTg) and 3xTgAD longitudinally (2, 4, 6, 12, and 15 months of age), we assessed the release of anti-inflammatory (IL-10), pro-inflammatory (TNFα, IL-1β, and IL-6), dual pro-inflammatory and regulatory (INFγ and IL-17), and regulatory (IL-2) cytokines in peritoneal leukocytes. The results of the release of the anti-inflammatory cytokine IL-10 both in the absence (resting condition) and in the presence of the mitogens concanavalin A (ConA) and lipopolysaccharide (LPS) are shown in Figure 1. Peritoneal leukocytes from 3xTgAD mice produced less IL-10 at 2 months of age than NTg without stimuli. In contrast, at 6 and 15 months of age, 3xTgAD mice released more significant amounts of IL-10 without stimulus than controls. No significant differences were observed in the presence of mitogens. When levels in the presence of mitogens were related to basal levels, to calculate the stimulation produced by mitogens on IL-10 release, we observed overstimulation of IL-10 production at 2 months and a lower stimulation of its production at 15 months in 3xTg mice compared to NTg mice with both mitogens. Concerning changes with age, we observed in both groups an increase in the release of this cytokine up to 6 months of age and a drastic decrease in its values at 12 months of age (Figure 1 and Supplementary Figure S1).

**Figure 2:**
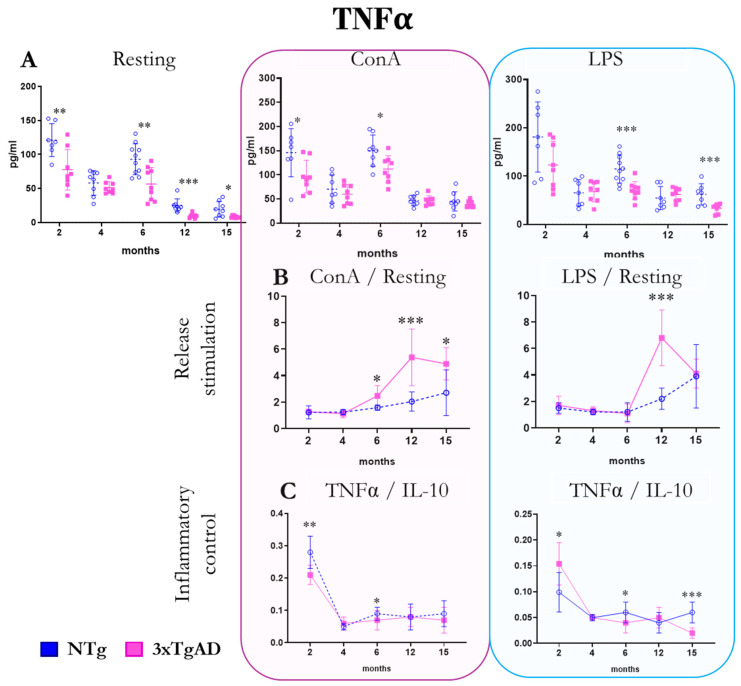
Changes with age in the release of the pro-inflammatory cytokine tumor necrosis factor-alpha (TNFα) by peritoneal leukocytes from non-transgenic (NTg) and triple-transgenic mice for Alzheimer’s disease (3xTgAD). (**A**): In the absence of stimulation (resting condition) and in the presence of the mitogens concanavalin A (ConA) and lipopolysaccharide (LPS). (**B**): Stimulation release (relating values in the presence and absence of mitogenic stimulus). (**C**): TNFα/IL-10 ratio. * *p* ≤ 0.05, ** *p* ≤ 0.01, *** *p* ≤ 0.001, differences between NTg and 3xTgAD.

The release of the pro-inflammatory cytokine TNFα by peritoneal leukocytes from NTg and 3xTgAD mice in the absence (resting condition) and the presence of mitogens (ConA and LPS) are shown in Figure 2. Without stimulus, 3xTgAD mice release lower amounts of TNFα at 2, 6, 12, and 15 months compared to NTg. In the presence of ConA, peritoneal leukocytes from 3xTgAD mice continue to release lower amounts of the cytokine at 2 and 6 months compared to NTg control mice. As for TNFα release in the presence of LPS, we observed lower amounts of this cytokine in 3xTgAD mice compared to NTg mice at 6 and 15 months. Regarding the stimulation of TNFα release by mitogens, mitogen condition/resting condition ratio, we appreciated an over-release of this cytokine at 6, 12, and 15 months in the presence of ConA and at 12 months in the presence of LPS in 3xTgAD mice compared to NTg. The pro-inflammatory response to mitogens was calculated by dividing the concentration of the cytokine TNFα by IL-10 values. In the presence of ConA, we observed a lower pro-inflammatory response at 2 and 6 months in 3xTgAD mice concerning NTg. In the presence of LPS, the pro-inflammatory response in 3xTgAD mice was higher at 2 months and lower at 6 and 15 months, relating to NTg. In relation to changes with age, the highest release of this cytokine is observed at 2 and 6 months of age (Figure 2 and Supplementary Figure S2).

**Figure 3:**
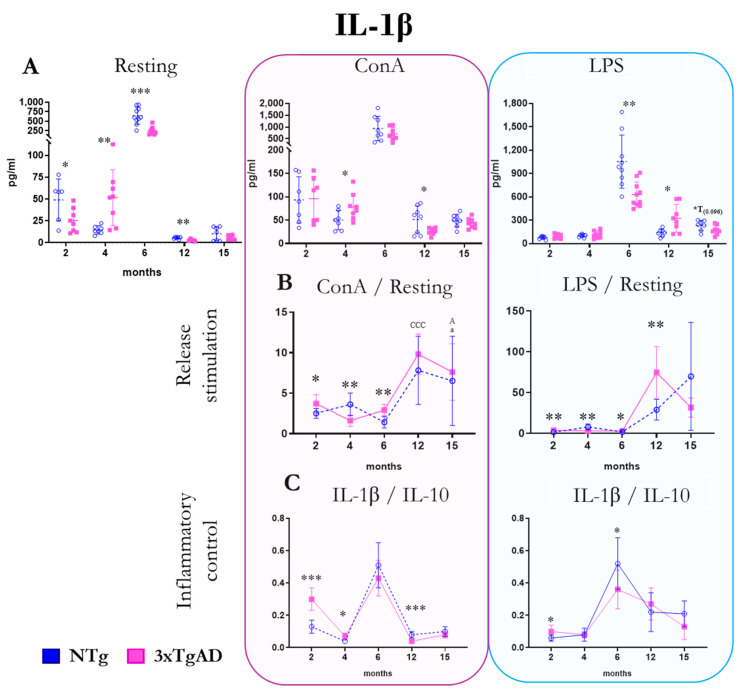
Changes with age in the release of the pro-inflammatory cytokine interleukin one beta (IL-1β) by peritoneal leukocytes from non-transgenic (NTg) and triple-transgenic mice for Alzheimer’s disease (3xTgAD) with age. (**A**): In the absence of stimulation (resting condition) and in the presence of the mitogens concanavalin A (ConA) and lipopolysaccharide (LPS). (**B**): Stimulation release (relating values in the presence and absence of mitogenic stimulus). a *p* ≤ 0.05, A *p* ≤ 0.05, CCC *p* ≤ 0.001. (**C**): IL-1β/IL-10 ratio. * *p* ≤ 0.05, ** *p* ≤ 0.01, *** *p* ≤ 0.001, differences between NTg and 3xTgAD.

The results of the release of the pro-inflammatory cytokine IL-1β by peritoneal leukocytes from NTg and 3xTgAD mice in the absence (resting condition) and the presence of mitogens (ConA and LPS) are shown in Figure 3. Under basal conditions, we observed lower IL-1β release at 2, 6, and 12 months and higher at 4 months in 3xTgAD mice compared to NTg mice. In the presence of ConA, we observed a higher release of this cytokine at 4 months and a lower release at 6 and 12 months in 3xTgAD mice concerning controls. In the case of cultures in the presence of LPS, a lower release of IL-1β was observed at 6 months and higher at 12 months in 3xTgAD concerning NTg. Regarding the stimulation of IL-1β release by mitogens, mitogen/basal ratios, a lower release of this cytokine is seen at 4 months and a higher one at 2 and 6 months in both conditions (with ConA and LPS) in 3xTgAD mice vs. controls. In addition, at 12 months, greater stimulation in the presence of LPS was observed in the 3xTgADs. We observed that the pro-inflammatory response (IL-1β/IL-10 ratio) in the presence of ConA was exacerbated at 2 and 4 months and was lower at 12 months in 3xTgAD mice compared to NTg. In the case of LPS, the IL-1β/IL-10 ratio was higher in 3xTgADs than in NTg at 2 months and lower at 6 months. Regarding changes with age, this cytokine shows a clear peak in its release by peritoneal leukocytes at 6 months of age (Figure 3 and Supplementary Figure S3).

**Figure 4:**
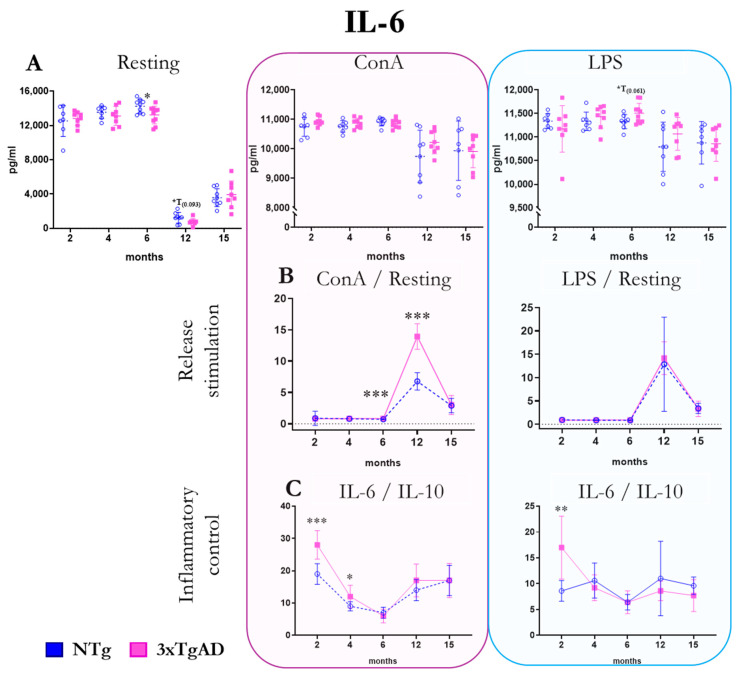
Changes with age in the release of the pro-inflammatory cytokine interleukin six (IL-6) by peritoneal leukocytes from non-transgenic (NTg) and triple-transgenic (3xTg) mice for Alzheimer’s disease (3xTgAD) mice with age. (**A**): In the absence of stimulation (resting condition) and in the presence of the mitogens concanavalin A (ConA) and lipopolysaccharide (LPS). (**B**): Stimulation release (relating values in the presence and absence of mitogenic stimulus). (**C**): IL-6/IL-10 ratio. * *p* ≤ 0.05, ** *p* ≤ 0.01, *** *p* ≤ 0.001, differences between NTg and 3xTgAD.

The release of the pro-inflammatory cytokine IL-6 by peritoneal leukocytes from NTg and 3xTgAD mice in the absence (resting condition) and the presence of mitogens (ConA and LPS) are shown in Figure 4. Under basal conditions, we observed a lower release of IL-6 at 6 months by peritoneal leukocytes from 3xTgAD mice relative to NTg mice. In the presence of mitogens, no significant differences were observed between Alzheimer’s mice and their respective controls. Regarding the stimulation of IL-6 release by mitogens and mitogen/basal ratios, we observed a higher stimulus with ConA at 6 and especially at 12 months in 3xTgAD mice compared to NTg. As for the pro-inflammatory response (IL-6/IL10 ratio), we observed that it is significantly higher in 3xTgAD mice than in controls at 2 and 4 months with ConA and at 2 months with LPS. In the absence of stimulus, a clear decrease in IL-6 release is observed from 12 months of age in both groups (Figure 4 and Supplementary Figure S4).

**Figure 5:**
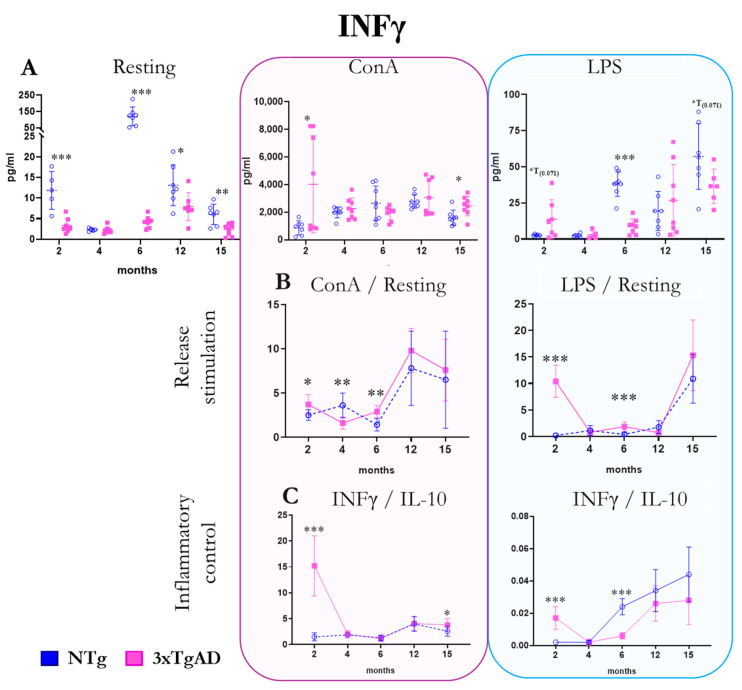
Changes with age in the release of the pro-inflammatory and regulatory cytokine interferon-gamma (INFγ) by peritoneal leukocytes from non-transgenic (NTg) and triple-transgenic mice for Alzheimer’s disease (3xTgAD) with age. (**A**): In the absence of stimulation (resting condition) and in the presence of the mitogens concanavalin A (ConA) and lipopolysaccharide (LPS). (**B**): Stimulation release (relating values in the presence and absence of mitogenic stimulus). (**C**): INFγ/IL-10 ratio. * *p* ≤ 0.05, ** *p* ≤ 0.01, *** *p* ≤ 0.001, differences between NTg and 3xTgAD.

The release of the pro-inflammatory/regulatory cytokine INFγ by peritoneal leukocytes from NTg and 3xTgAD mice in the absence (resting condition) and the presence of mitogens (ConA and LPS) are shown in Figure 5. Under basal conditions, we observed a lower release of this cytokine at 2, 6, 12, and 15 months in 3xTgAD mice with respect to NTg mice. In the presence of ConA, a higher release was observed at 2 and 15 months, and in the presence of LPS, this release was lower at 6 months in 3xTgAD mice compared to controls. Regarding the stimulation of INFγ release by mitogens, mitogen/basal ratios, we observed higher in 3xTgAD mice vs. NTg mice in the presence of the ConA mitogen at 2, 6, and 15 months and 2 and 6 months in the presence of LPS. Concerning the pro-inflammatory response (INFγ/IL10 ratio), we observed an exacerbated response at 2 months in both ConA and LPS and a lower response at 6 months with LPS in 3xTgAD mice compared to controls. Changes in the age of the INFγ will depend on the group being studied. While in control mice, we observed the highest release of INFγ in the absence of stimulus at 6 months of age; in the case of 3xTgAD mice, this occurs at 2 months of age in the presence of mitogens (Figure 5 and Supplementary Figure S5).

**Figure 6:**
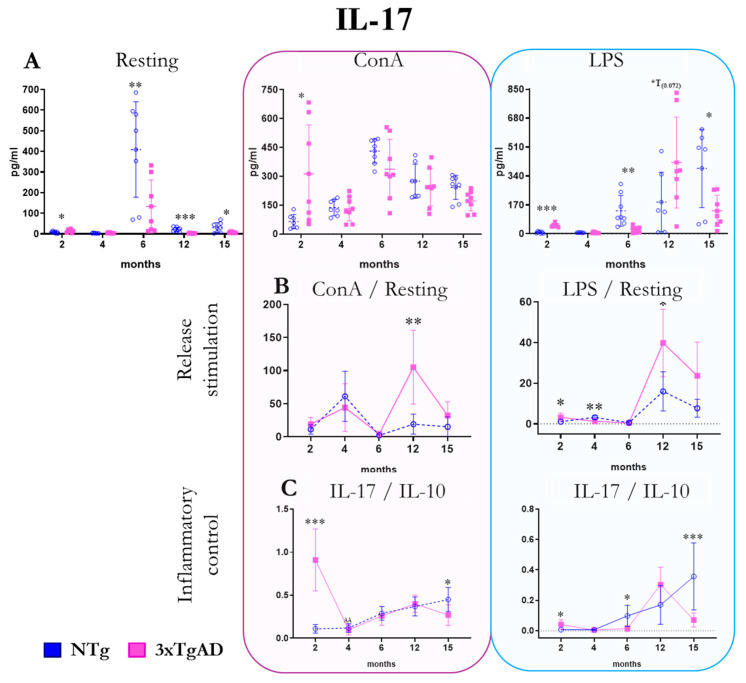
Changes with age in the release of the pro-inflammatory and regulatory cytokine interleukin seventeen (IL-17) by peritoneal leukocytes from non-transgenic (NTg) and triple-transgenic mice for Alzheimer’s disease (3xTgAD) with age. (**A**): In the absence of stimulation (resting condition) and in the presence of the mitogens concanavalin A (ConA) and lipopolysaccharide (LPS). (**B**): Stimulation release (relating values in the presence and absence of mitogenic stimulus). (**C**): IL-17/IL-10 ratio, AA *p* ≤ 0.01. * *p* ≤ 0.05, ** *p* ≤ 0.01, *** *p* ≤ 0.001, differences between NTg and 3xTgAD.

The release of the pro-inflammatory/regulatory cytokine IL-17 by peritoneal leukocytes from NTg and 3xTgAD mice in the absence (resting condition) and the presence of mitogens (ConA and LPS) are shown in Figure 6. Under basal conditions, higher release is shown at 2 months and lower release from 6 months onwards in 3xTgAD mice compared to control mice. When the culture is stimulated with ConA, a higher release of IL-17 is observed at 2 months in 3xTgAD mice compared to NTg mice, and when stimulated with LPS, there is also a higher release at 2 months, but a lower release appears at 6 and 15 months. Regarding the stimulation of IL-17 release by mitogens and mitogen/basal ratios, we observed overstimulation of the release of this cytokine at 12 months in the presence of ConA in 3xTgAD mice with respect to NTg. With LPS, this is shown at 2 and 12 months, but there is less stimulation at 4 months. Regarding the pro-inflammatory response (IL-17/IL10 ratio), we observed a response in 3xTgAD mice concerning NTg, with both mitogens higher at 2 months and lower at 15 months. Moreover, this lack of stimulation in IL-17 production with respect to IL-10 was also observed at 6 months in 3xTgAD mice compared to NTg in response to LPS. Changes in IL-17 with age show an increase in its release up to 6 months and a subsequent decrease. This increase is more significant without stimulus (Figure 6 and Supplementary Figure S6).

**Figure 7:**
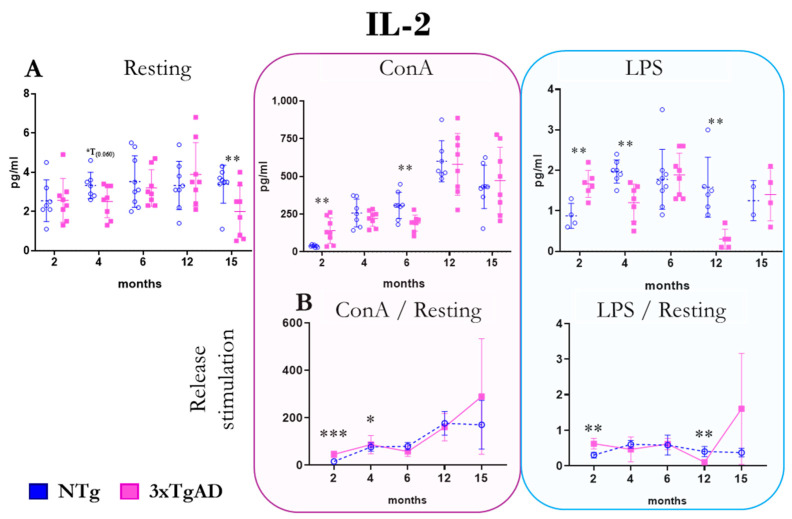
Changes with age in the release of the regulatory cytokine interleukin two (IL-2) by peritoneal leukocytes from non-transgenic (NTg) and triple-transgenic mice for Alzheimer’s disease (3xTgAD). (**A**): In the absence of stimulation (resting condition) and in the presence of the mitogens concanavalin A (ConA) and lipopolysaccharide (LPS). (**B**): Stimulation release (relating values in the presence and absence of mitogenic stimulus). * *p* ≤ 0.05, ** *p* ≤ 0.01, *** *p* ≤ 0.001, differences between NTg and 3xTgAD.

The release of the regulatory cytokine IL-2 by peritoneal leukocytes from NTg and 3xTgAD mice in the absence (resting condition) and the presence of mitogens (ConA and LPS) are shown in Figure 7. At 12 months, 3xTgAD mice released more of this cytokine without stimulus than NTg mice. In the presence of ConA, peritoneal leukocytes from 3xTgAD mice released more IL-2 at 2 months and less at 6 months relative to controls. As for IL-2 release in the presence of LPS, 3xTgAD mice also released more IL-2 at 2 months and less at 4 and 12 months compared with NTg mice. Regarding the stimulation of IL-2 release by mitogens and mitogen/basal ratios, higher values are observed in the presence of ConA at 2 and 4 months in 3TgAD than in controls. In the presence of LPS, there are also higher values at 2 months but lower values at 12 months in 3xTgAD mice compared to NTg. Changes in IL-2 release with age depend on the type of stimulus received, but the highest release occurs around 6 months of age (Figure 7 and Supplementary Figure S7).

### 2.2. Oxidative Stress

**Figure 8:**
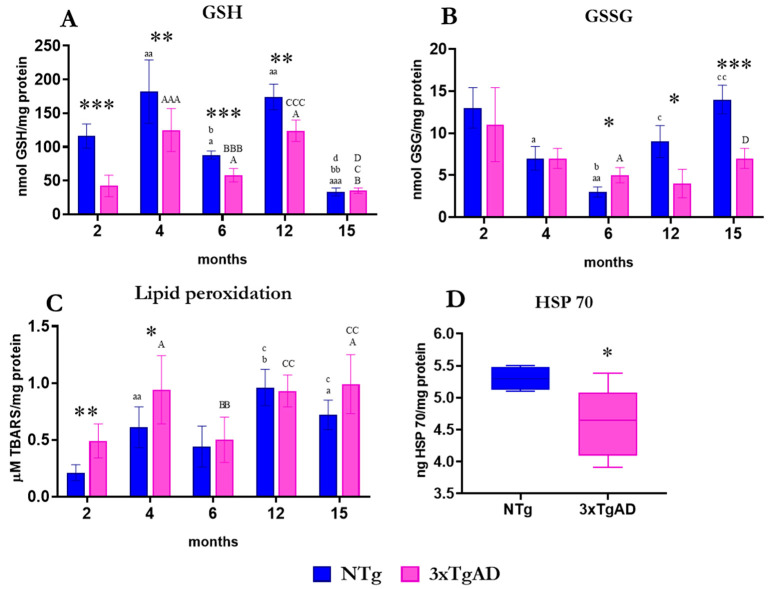
Oxidative stress markers in peritoneal leukocytes from non-transgenic (NTg) and triple-transgenic mice for Alzheimer’s disease (3xTgAD). (**A**) Reduced glutathione (GSH). (**B**) Oxidized glutathione (GSSG). (**C**) Thiobarbituric acid reactive species (TBARS) concentrations as a marker of lipid peroxidation. (**D**) Heat shock protein 70 (Hsp70) concentrations as a marker of misfolded proteins. Differences between NTg and 3xTgAD are represented by * *p* ≤ 0.05, ** *p* ≤ 0.01, *** *p* ≤ 0.001. Differences within the NTg group with age defined by a *p* ≤ 0.05, aa *p* ≤ 0.01, aaa *p* ≤ 0.001 vs. 2 months; b *p* ≤ 0.05, bb *p* ≤ 0.01 vs. 4 months; c *p* ≤ 0.05, cc *p* ≤ 0.01 vs. 6 months. Differences within the 3xTgAD group with age represented by A *p* ≤ 0.05, AAA *p* ≤ 0.001 vs. 2 months; B *p* ≤ 0.05, BB *p* ≤ 0.01, BBB *p* ≤ 0.001 vs. 4 months; C *p* ≤ 0.05, CC *p* ≤ 0.01, CCC *p* ≤ 0.001 vs. 6 months; D *p* ≤ 0.05 vs. 12 months.

The parameters analyzed as indicative of oxidative stress (Figure 8) showed a lower concentration of reduced glutathione (GSH), the main cellular antioxidant defense, in 3xTgAD mice than in NTg at most ages analyzed. However, the concentration of oxidized glutathione (GSSG), a biomarker of oxidation, at 6 months was higher in 3xTgAD compared to NTg, although lower at 12 and 15 months of age. The oxidative lipid damage (lipid peroxidation) shows a higher concentration of TBARS in the peritoneal leukocytes of 3xTgAD mice compared to NTg mice at 2 and 4 months. The amounts of Hsp70, an indirect marker for misfolded proteins, in adulthood (6 months) are lower in 3xTgAD mice than controls. 

### 2.3. Longevity

**Figure 9:**
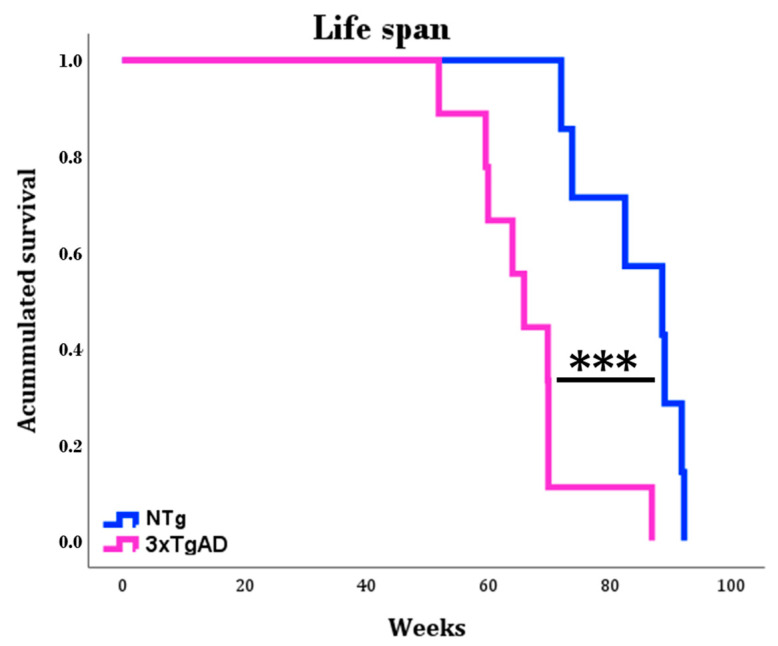
Survival curves of non-transgenic mice (NTg) and triple-transgenic mice for Alzheimer’s disease (3xTgAD). Differences between NTg and 3xTgAD are represented by *** *p* ≤ 0.001.

Finally, the survival (Figure 9) of both groups of animals was determined, and the 3xTgADs had a mean life expectancy (66.5 ± 3.2 weeks) significantly lower than that of the NTg (84.4 ± 3.2 weeks).

## 3. Discussion

In AD, brain damage is irreversible once clinical symptoms appear, making it difficult to find an effective treatment [46,47]. For this reason, it is imperative to improve our understanding of the pathophysiology of this disease, which has been investigated since its description in 1907 by Alois Alzheimer. There still needs to be a consensus on what the origin of the pathology is or could be. A fact is the involvement of brain glial cell-mediated inflammation in the pathogenesis of the disease [12,13]. However, peripheral inflammation, such as that produced by immune cells by releasing pro-inflammatory cytokines, may reach the brain and contribute to neuroinflammation. For example, in AD, monocytes will be recruited from the periphery to clear Aβ deposits [48]. Several possible biomarkers for AD onset and progression have been suggested in this context, most related to the immune system, inflammation, and oxidative stress, such as phagocytic capacity against Aβ [49], proliferative response, chemotaxis, cytokines (IL-1β, IL-2, IL-6, TNFα…), glutathione or glutathione regulatory enzymes, thiobarbituric acid reactive substances (TBARS), and malondialdehyde (MDA) [21,23,42,46,49,50,51,52,53,54,55]. Moreover, all these potential biomarkers are interrelated. Due to its function, the immune system is the primary source of cytokines and oxidative stress [18]. In this sense, despite evidence that there is an association between abnormal inflammation and Alzheimer’s disease and that this abnormal inflammation precedes the development of the disease, these biomarkers are not well established, underlining the complex nature of the disease and the challenge it poses for further research. 

In 3xTgAD animals, the disease begins to present manifestations, such as the appearance of extracellular β-amyloid plaques in the brain at six months of age. Subsequently, this extracellular deposition of Aβ that has started in the prefrontal cortex extends to the hippocampus and cortical regions, which is appreciable at 12 months in these animals. At this time, hyperphosphorylations of the tau protein begin [40,56], giving rise to significant neurodegeneration that correlates with behavioral deterioration [57]. At 15 months, these mice have fully established disease.

The present study is the first to evaluate, longitudinally, the progression of several inflammatory and oxidative stress parameters in 3xTgAD mice from 2 to 15 months of age compared with NTg controls. In general, young 3xTgAD mice have a marked pro-inflammatory response and oxidative stress at the peripheral level, even before immunoreactivity against Aβ appears at 2,5 months of age [39,40,42,57]. 

Previously, our group investigated how parameters of immune function and oxidative stress varied over time in peritoneal leukocytes from female 3xTgAD mice. We observed dysfunction in most of the immune functions studied. Additionally, we found lower antioxidant activity during the early stages of the disease, as well as decreased longevity [42].

Regarding the amounts of pro- and anti-inflammatory cytokines in patients with AD or cognitive impairment, studies have been conducted in cerebrospinal fluid and plasma, but the results are contradictory. Thus, in the plasma of patients with mild cognitive impairment, some results have shown an increase in some pro-inflammatory cytokines [21,22,58,59]. In contrast, others found no differences [59,60,61] or reduced amounts [59]. However, most investigations of AD patients showed an increase in plasma pro-inflammatory cytokines [22,62] and a correlation between pro-inflammatory cytokine levels and disease severity [62,63]. However, there are hardly any longitudinal studies on murine models.

In the present work, the results obtained for IL-10 show higher concentrations in basal cultures of cells from 3xTgAD mice than in NTg mice from 4 months onwards, reaching statistically significant differences at 6 and 15 months. This anti-inflammatory cytokine suppresses or controls the inflammatory response [8,64]. Although this role of IL-10 could be positive for the organism, several studies have linked increased IL-10 levels to an aggravation of the disease in mice [64,65,66]. Also, serum IL-10 concentration correlates with beta-amyloid deposition in cerebrospinal fluid of AD patients [67]. Thus, the higher amounts of IL-10 released at 6 and 15 months could reflect the disease progression. The lower amounts of IL-10 released by 3xTgAD peritoneal cells at 2 months may be either a reflection of the inability of these animals to adequately synthesize this protective cytokine or a depletion of it following their attempt to control inflammation already existing at the peripheral level at that age.

The most typical pro-inflammatory cytokine assessed in this work, TNF-α, is released in lower amounts by peritoneal cells of 3xTgAD mice, both at basal and in response to mitogens. However, when observing the stimulation of the release of this cytokine employing the ratio between its release in response to mitogens and basal, it is appreciated that these ratios are higher in 3xTgADs than in controls at ages when the disease is already more established. Previous studies showed a higher secretion of TNFα in splenic leukocytes in the presence of mitogens in old 3xTgAD mice compared to NTg [57,68,69]. It should be taken into account that this cytokine is fundamental in the pathogenesis of AD as it is secreted by microglia but also by peripheral immune cells and that it is involved in the permeability of intestinal and blood–brain barriers, which would favor neuroinflammation [70,71,72].

IL-1β is a crucial factor in the inflammatory response and modulates the expression of other cytokines, such as IL-6 or TNFα [11,64,73]. In the AD brain, this cytokine plays an essential role in Aβ plaque deposition and hyperreactivity of microglia and astrocytes [8,74]. At the peripheral level, it is elevated in patients with AD [75,76,77]. In the present work, in general, a lower release of this cytokine by peritoneal cells appears in 3xTgADs than in controls, both at the basal level and in response to mitogens. However, when the ratios between the release in the presence of mitogen and the basal release are analyzed, it is observed that at early ages, the values are higher in the transgenics than in the NTg. 

IL-6 is a pro-inflammatory cytokine that has also been found to be elevated in AD patients. In the present work, hardly any differences were found in the released concentrations of this cytokine between 3xTgADs and controls. In this regard, Taketa et al. [20] demonstrated, in a mouse model of Alzheimer’s disease, how peripheral injection of LPS increased TNFα and IL-6 concentrations in the brain, as well as it did in the permeability of the blood–brain barrier and how it entailed behavioral impairment. Moreover, all this occurred without any change in the plasma levels of these cytokines. 

IFNγ is an activator of monocyte function that engages in the inflammatory response [64] and has been linked to neuropsychiatric symptoms associated with AD [21,78,79]. The results of this marker are contradictory among the different studies analyzed, with some pointing to a significant increase in patients with MCI [21] and others to a substantial increase in patients with AD [80]. 

Even though IL-17 is considered a regulatory cytokine, it has been proven to act pro-inflammatory in the autoimmune process [68]. Also, it is released in lower amounts by leukocyte cells from 3xTgADs than from NTg in the basal state, although this takes place or the opposite in response to mitogens, depending on the age of the animals. 

IL-2 is a regulatory cytokine and is an essential activator of proliferation and NK activity. It has also been pro-inflammatory in certain situations [81,82]. In the present work, we have observed that 3xTgAD animals have a much higher release of this cytokine in response to mitogens than NTg at 2 months, with the opposite occurring at older ages. In this regard, patient results are controversial, as some show an increase [62] while others show no change [83].

It is worth noting that some cytokines undergo age-dependent changes in this study, like IL-6, whose levels are initially elevated at younger ages and then decrease, or IL-1β, INF-γ, and IL-17 concentrations increase at 6 months. The changes in inflammatory stress with age, especially at early ages, have been studied mainly in humans. These studies show great variability due to factors such as age or the technique and type of sample used. Thus, it has been shown that cytokines such as IL-6, IL-1β, and INF-γ show a marked increase in their production/release in adolescence or early adulthood, similar to that observed in the present work. Most studies indicate that increased pro-inflammatory cytokines during childhood could impact crucial development [84,85,86,87,88,89].

One of the most relevant results obtained in the present study is the exacerbation of the immune response of peritoneal leukocytes of 3xTgAD mice when confronted with a mitogen concerning the release of these cytokines without stimuli, especially at 2 months of age when there is no disease symptomatology. This alteration could be implicated in the increased permeability of the blood–brain barrier and the induction of changes in the brain, such as microglia activation or Aβ production [20,90,91]. Pro-inflammatory cytokines would play a priori, an antimicrobial effect [92,93]. Still, as this is a significant response compared to controls, it could be responsible for damage to different tissues and, specifically, to the brain and could be critical in the pathogenesis of the disease. Evidence shows how peripheral inflammation induces neuroinflammation and brain damage [94,95,96,97]. Likewise, it is known that inflammation at the brain level can cause systemic inflammation that will ultimately enhance neuroinflammation and brain damage. In this sense, brain damage will lead to the infiltration of immune cells into the central nervous system, enhancing neuroinflammation and peripheral inflammation [96,97,98,99]. In this sense, it is worth mentioning that there is constant communication between the nervous and immune systems, called neuroimmunoendocrine communication, due to the presence of receptors for the mediators of the other system in each of them [18,100,101,102,103].

The exacerbated response to stimulus observed in this study supports the infection theory of Alzheimer’s disease [4,25]. Thus, when faced with a stimulus such as an infection, the peripheral immune cells of individuals with AD would respond disproportionately and, therefore, would end up causing damage to the individual. This is supported by the most recent studies that postulate that Aβ is an antimicrobial that can function as a cytokine. This exacerbated response is also observed in autoimmune pathologies, where a disproportionate response causes damage. Accordingly, both autoimmune diseases and AD are more frequent in women, and both processes have been related to a dysregulation of the immune system. In addition, these immune alterations and their appearance have been linked to estrogen concentrations, particularly the role of estrogen loss during menopause [104,105,106].

Regarding the oxidative stress parameter analyzed in the present work, we have observed in peritoneal leukocytes from 3xTgAD mice, in comparison to those of NTg, a lower amount of a relevant antioxidant defense such as the GSH, even at the earliest ages, before the first alterations appear in the brain. However, the GSSG concentration was higher. This oxidative stress is consistent with what we had previously observed in these peritoneal leukocytes at early ages [42]. This oxidative stress could explain the higher oxidized lipids detected in the present study, which showed higher lipid peroxidation in peritoneal cells of 3xTgAD mice compared to NTg at 2 and 4 months. Similar results have been obtained in brain slices of 3xTgAD mice with increased lipid peroxidation before 6 months [107]. This increased amount has also been observed in neutrophils [23] and blood cells [52] from individuals with mild cognitive impairment and Alzheimer’s disease. Moreover, it should be considered that this higher lipid peroxidation constitutes a relevant oxidative potential that can affect the rest of the organism [108].

In addition, we observed in these 3xTgAD mice a decrease in Hsp70 protein at 6 months. This protein protects cells undergoing stress by binding and processing unfolded, misfolded, or aberrant proteins [109]. Not only that, but Hsp70 is critical for cellular homeostasis as it regulates functions of protein folding and transport, apoptosis, and inflammation [110]. Adequate production of this protein protects the peripheral level from oxidative stress and inflammation. At the brain level, it exerts neuronal protection, synaptic plasticity, and memory consolidation [111]. The decreased presence of this protein in the cells of 3xTgAD mice could indicate a loss of this cellular homeostasis in the peritoneal leukocytes of 3xTgAD mice, similar to what happens with aging [112]. 

Finally, in the present study, we observed lower longevity in 3xTgAD mice compared to NTgs mice, corroborating previous results of our group with these animals [42]. It is again shown that impairment of the regulatory, nervous, endocrine, and immune systems, as in these 3xTgAD mice, results in a shorter life expectancy [57,69]. In this regard, states with high inflammatory and oxidative stress have been related to increased mortality [113,114,115,116,117,118,119]. In recent years, it has been observed that the relationship of pro-inflammatory cytokines with anti-inflammatory compounds at the peripheral level, particularly the IL-10/TNF ratio, indicates successful aging and longevity [120,121], where higher ratios positively correlate with increased lifespan. 

The results of the present work suggest that 3xTgAD mice show inflammatory stress in their peritoneal leukocytes and increased oxidative stress and peroxidative damage in the prodromal phase of the disease compared to control animals. However, these differences seem to be disappearing with advancing age, possibly due to a more significant release of anti-inflammatory cytokines such as IL-10, as has been proven in this work, but also by becoming equal in the oxidative state with controls due to advancing age [42]. The early oxidation-inflammation of 3xTgADs that appears at the peripheral level could be the basis of the development of the disease and its lower longevity. In addition, the fact that inflammatory and oxidative stress alterations occur before the establishment of the disease makes them promising biomarkers for the early arrest of the disease. Not only that, but they could be suitable therapeutic targets. In this sense, there has been increased interest in basic and clinical studies on the possible therapeutic role of antioxidants in AD [122,123,124,125]. 

## 4. Materials and Methods

### 4.1. Animals

Female triple-transgenic mouse models for ×AD (3xTgAD, *n* = 8) and non-transgenic (NTg, *n* = 7) wild-type 129/C57BL6 were obtained from Dra’s laboratory Lydia Giménez-Llort from the Department of Psychiatry and Forensic Medicine at the Institute of Neurosciences of the Autonoma University of Barcelona (Bellaterra, Spain) at the age of 1.5 months. Females were chosen over males given the ease of caging them together, as males show aggressive and hierarchic behavior when housed together, which results in scars that tremendously affect inflammatory responses. All the mice were housed in 4–5 per cage and maintained in standard laboratory animal conditions, with temperature (22 ± 2 °C) and humidity (50–60%) on a 12/12 h reversed light/dark cycle (lights on at 20:00 h) to avoid circadian interferences. Mice had access to tap water and standard pellets *ad libitum*. Diet was under the recommendations of the American Institute of Nutrition for laboratory animals.

### 4.2. Experimental Design

**Figure 10:**
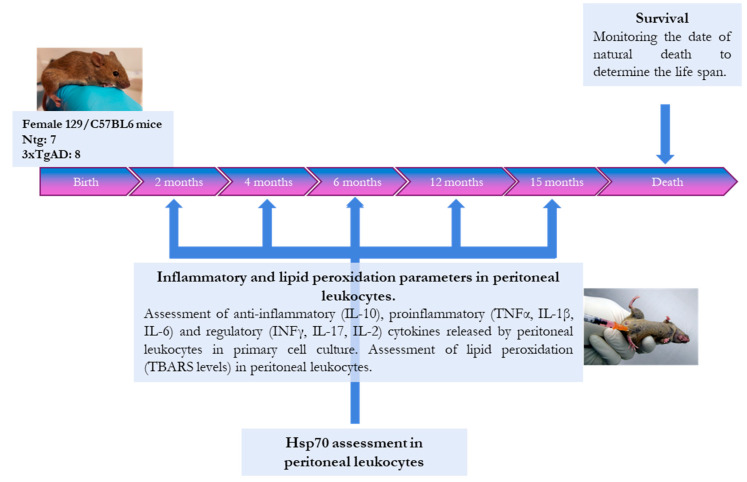
The study’s experimental design analyzes oxidative and inflammatory stress in triple-transgenic mice for Alzheimer’s disease. Abbreviations: IL: interleukin; INF: interferon; Hsp70, heat shock protein 70; Ntg, non-transgenic mice; TBARS, thiobarbituric acid reactive substances; TNF, tumor necrosis factor; 3xTgAD, triple-transgenic mice for Alzheimer’s disease.

The experimental design of the present study is shown in Figure 10. To assess the inflammatory and oxidative status of 3xTgAD mice, peritoneal leukocytes were extracted from mice at 2, 4, 6, 12, and 15 months of age. These peritoneal leukocytes were used to make cell cultures to assess inflammatory status, and pellets of the leukocytes were stored to evaluate oxidative stress. In addition, animals were monitored until natural death to determine longevity. As inflammatory stress parameters, the anti-inflammatory cytokine IL-10, the pro-inflammatory cytokines TNFα, IL-1β, and IL-6, the dual function cytokines that can function as pro-inflammatory or regulatory INFγ and IL-17, and the regulatory cytokine IL-2 were assessed. As oxidative stress parameters, lipid peroxidation was evaluated by determining thiobarbituric acid reactive substances (TBARSs). In addition, the amount of heat shock protein 70 was analyzed in adulthood (6 months) as a marker of misfolded protein accumulation.

### 4.3. Collection of Peritoneal Leukocytes

Leukocyte suspension was obtained from the peritoneal cavity at 2 ± 1, 4 ± 1, 6 ± 1, 12 ± 1, and 15 ± 1 months of age. The collection was performed between 8:00 and 10:00 to avoid circadian variations. This technique is minimally invasive without the requirement of sacrificing the animals. Non-anesthetized mice were held by cervical skin, the abdomen was cleaned with 70% ethanol, and 3ml of sterile Hanks’s solution at 37 °C was injected intraperitoneally. A total of 80–90% of the injected volume was recovered by abdominal massage. Neubauer chambers quantified the peritoneal suspensions, and the concentration was adjusted for each test in a complete medium containing 1640RPMI (PAA, Piscataway, NJ, USA) supplemented with gentamicin (10m g/mL, PAA) and 10% heat-inactivated fetal calf serum (PAA).

### 4.4. Cytokine Measurement

Cytokine production was assessed in peritoneal leukocyte cultures under three distinct conditions: absence of stimuli (basal condition) and in the presence of mitogens concanavalin A (ConA, 1 µg/mL, Sigma-Aldrich, St. Louis, MO, USA) and bacterial lipopolysaccharide (LPS, 1 µg/mL, Sigma-Aldrich). For this purpose, 200 µL of the adjusted leukocyte suspension aliquots (1 × 10^6^ lymphocytes/mL) were dispensed into 96-well plates (Nunc, Roskilde, Denmark) with 20 µL of complete medium, ConA, or LPS. Each condition was performed in triplicate. After 48 h of incubating at 37 °C in a sterile and humidified atmosphere of 5% CO_2_, 100 µL of the culture supernatant was collected and stored at −80 °C until its use.

The levels of TNF-α, IFNγ, IL-1β, IL-2, IL-6, IL-17, and IL-10 were measured simultaneously by multiplex luminometry using a mouse cytokine/chemokine panel (Milliplex Map Kit MCYTOMAG-70K, Merck, Darmstadt, Germany). After pre-wetting the plate with assay buffer, we added 25 µL of standards, controls, or experimental samples to the appropriate wells and 25 µL of premixed bread in each well. After overnight incubation at 4 °C with shaking and three washes, 25 µL of detections antibody was added to each well and incubated for 1 h at room temperature on a plate shaker. Subsequently, 25 µL of Streptavidin–Phycoerythrin was added and incubated with shaking for another 30 min at room temperature. The plate was then washed trice with wash buffer and vacuum filtered, and, finally, the beads were resuspended in 150 µL of sheath fluid, and the plate was read using a luminometer (Luminex-200, Luminex Corporation, Austin, TX, USA). The results were expressed as pg/mL. Concentrations as low as 2.3 pg/mL for TNFα, 1.1 pg/mL for INFγ, 5.4 pg/mL for IL-1β, 1.0 pg/mL for IL-2, 1.1 pg/mL for IL-6, 0.5 pg/mL for IL-17, and 0.8 pg/mL for IL-10 can be detected.

In addition, the stimulation’s ability to produce pro-inflammatory, regulatory, and anti-inflammatory cytokines in the presence of stimulus was assessed by dividing the stimulation concentration by the basal concentration. Finally, the control of the pro-inflammatory response was evaluated by dividing the values obtained for the pro-inflammatory cytokines by the basal concentration.

### 4.5. Oxidative Damage

Oxidative damage was assessed by quantifying the amount of oxidized and reduced glutathione, thiobarbituric acid reactive substances (TBARS) as a marker of lipid damage [126,127] and by quantifying the chaperone heat shock protein 70 (HSP70), whose production is induced by protein damage [128]. For glutathione and TBARS levels, 1 × 10^6^ cells/mL pellets were obtained at the above ages. For HSP70 levels, 3 × 10^6^ cells/mL pellets were obtained at 6 months, when the disease is fully established. The pellets were obtained by centrifugation of the samples at 1 × 100 g for 10 min and frozen at −80 °C until their use.

For the determination of the levels of both forms of glutathione (oxidized and reduced), a modification [42] of the method of Hissin and Hilf [129], adapted to be carried out in plate by using the fluorescent probe O-phthaldialdehyde (OPT), was used. The method is based on the ability of GSSG at pH 12 and GSH at pH 8 to react with OPT, leading to the formation of a fluorescent compound that is activated at a wavelength (λ) of 350 nm and which presents a maximum emission point at λ = 420 nm. After obtaining the sediment of 106 peritoneal leukocytes, it was resuspended in 100 mM phosphate buffer containing 50 mM EDTA, pH 8. Subsequently, the cells were coldly sonicated (3 cycles of 10 s with 20 s of rest between cycles), and 5 μL of 60% HClO_4_ was added. Next, the sample was centrifuged at 9500× *g* for 10 min at 4 °C. A total of 10 μL of the supernatant was dispensed into two black 96-well flat-bottom plates, one for each form of glutathione. For GSSG measurement, 12 μL of *n*-ethylmaleimide (NEM, 0.04 M) was added to each well to prevent the transformation of GSH to GSSG that takes place at pH 8, and the plate was incubated in the dark at room temperature for 30 min. Subsequently, 178 μL of NaOH (0.1 *n*), pH 12.5, and 20 μL of OPT (1mg/mL in methanol) were added and incubated for 15 min under the same conditions above. Finally, the fluorescence emitted by each well was determined at a wavelength of 350 nm excitation and 420 nm emission. To measure GSH content, 190 μL of phosphate buffer (100 mM with 50 mM EDTA, pH 8) and 20 μL of OPT were added to the 10 μL supernatant. After a 15-min incubation under the same conditions, the fluorescence emitted by each well was measured at the same wavelengths. Results were expressed as nmol of GSSG or GSH per milligram of protein or million cells.

Oxidative damage was assessed by quantifying the amount of thiobarbituric acid reactive substances (TBARS) as a marker of lipid damage [126,127] and by quantifying the chaperone heat shock protein 70 (HSP70), whose production is induced by protein damage [128]. For TBARS levels, 1 × 10^6^ cells/mL pellets were obtained at the above ages. For HSP70 levels, 3 × 10^6^ cells/mL pellets were obtained at 6 months, when the disease is fully established. The pellets were obtained by centrifugation of the samples at 1 × 100 g for 10 min and frozen at −80 °C until their use.

The procedure used for the determination of TBARS concentration was performed using the “Lipid Peroxidation Assay Kit” (Biovision, San Francisco, CA, USA) as previously described [130,131]. Briefly, 106 cells/mL of Hank’s solution were dispensed into Eppendorf, a 10-min wash was performed at 1100 g, and the supernatant was removed. Aliquots were resuspended in 200 μL of lysis buffer, and samples were centrifuged at 3200× *g* for 30 min. After centrifugation, the supernatant of the samples (0.2 mL) was collected in an Eppendorf tube protected with aluminum foil. Both these Eppendorf tubes and the standard curve tubes were spiked with 0.6 mL of thiobarbituric acid (TBA), and the center of the cap was punctured and incubated for 1 h at 95 °C. After the incubation, they were cooled for 10 min on ice, 0.3 mL of butanol was added and centrifuged at 1700× *g* for 15 min at 4 °C. Finally, the organic phase (0.2 mL) was collected and loaded into a 96-well flat-bottom plate. Once the plate was loaded, absorbance was measured at 532 nm. The results were expressed as micromole TBARs per milligram of protein.

The HSP70 levels were assessed by the “Hsp70 ELISA Kit” (ADI-EKS-700B, Enzo Life Sciences, Farmingdale, NY, USA) as previously described [110,112]. Briefly, 3 × 10^6^ pellets resuspended in extraction buffer were sonicated with 3 pulses (level 7, Sonic Dismembrator 60, Fisher Scientific, Hampton, NH, USA) after 30 min incubation on ice. Subsequently, the samples were centrifugated for 30 min at 20,000× *g*, and the supernatants were diluted 4-fold in Sample Dilutent 2 provided in the kit. At room temperature, 100 µL of standards and samples were incubated in the anti-Hsp70 immunoassay plate for 2 h. The plate was washed four times before a 1-h incubation with 100 µL of Hsp70 antibody in each well at room temperature. After washing the plate four times, 100 µL of Hsp70 conjugate was added to each well and incubated for 1h at room temperature. Subsequently, 100 µL of TMB substrate was added to each well and incubated for another 30 min at room temperature. Finally, 100 µL of stop solution was added, and the absorbance was read at 450 nm. The results were expressed as nanograms of HSP70 per milligram of protein.

### 4.6. Protein Quantification

Protein quantification was performed using the bicinchoninic acid (BCA) method, using the “BCA kit” (Merck, Darmstadt, Germany). This method is based on reducing Cu^2+^, generating Cu^+^ ions that bind to BCA and form a colored compound that absorbs light at 562 nm. Protein assessment was carried out on the same supernatants collected from the analysis of the TBARs and HSP70 as previously described [131]. The results are expressed in milligrams of protein per milliliter.

### 4.7. Statistical Analysis

Statistics were performed using SPSS version 28.0 (Chicago, IL, USA). The Kolmogorov–Smirnov test analyzed the normality of the samples. One-way ANOVA evaluated the differences between groups at each age and the t-Student. The significance level of *p* ≤ 0.05 was considered the minimum significance level.

## Data Availability

Data are contained within the article.

## Supplementary Materials

The following supporting information can be downloaded at: https://www.mdpi.com/article/10.3390/ijms25136976/s1.
