# Supplementary material for: Prodromic Inflammatory–Oxidative Stress in Peritoneal Leukocytes of Triple-Transgenic Mice for Alzheimer’s Disease
Source: Int J Mol Sci. 2024 Jun 26;25(13):6976. doi: 10.3390/ijms25136976 (PMC11241217; doi:10.3390/ijms25136976)
Supplement: Supplementary file 1 [file ijms-25-06976-s001.zip › Supplementary Materials.pdf]

# IL-10

A

| Resting | NTg | | | | |
|---|---|---|---|---|---|
| 3xTgAD | 2 | 4 | 6 | 12 | 15 |
| 2 | | - | * | ** | - |
| 4 | - | | ** | ** | - |
| 6 | * | * | | *** | - |
| 12 | ** | *** | ** | | - |
| 15 | - | - | - | - | |

| ConA | NTg | | | | |
|---|---|---|---|---|---|
| 3xTgAD | 2 | 4 | 6 | 12 | 15 |
| 2 | | * | * | - | - |
| 4 | * | | - | - | * |
| 6 | * | - | | ** | *** |
| 12 | * | * | *** | | - |
| 15 | - | * | *** | - | |

| LPS | NTg | | | | |
|---|---|---|---|---|---|
| 3xTgAD | 2 | 4 | 6 | 12 | 15 |
| 2 | | - | * | - | - |
| 4 | - | | - | - | - |
| 6 | * | - | | - | - |
| 12 | - | - | - | | - |
| 15 | * | - | - | - | |

B

| ConA / Resting | NTg | | | | |
|---|---|---|---|---|---|
| 3xTgAD | 2 | 4 | 6 | 12 | 15 |
| 2 | | * | - | * | - |
| 4 | - | | ** | * | - |
| 6 | - | - | | ** | * |
| 12 | * | * | * | | - |
| 15 | - | - | * | - | |

| LPS / Resting | NTg | | | | |
|---|---|---|---|---|---|
| 3xTgAD | 2 | 4 | 6 | 12 | 15 |
| 2 | | - | ** | ** | - |
| 4 | * | | ** | * | * |
| 6 | ** | * | | * | * |
| 12 | - | ** | * | | - |
| 15 | - | * | ** | * | |

**Figure S1.** Statistical differences of the age-dependent changes in the release of the anti-inflammatory cytokine interleukin ten (IL-10) by peritoneal leukocytes from non-transgenic (NTg) and triple transgenic (3xTg) mice for Alzheimer's disease (3xTgAD). A: In the absence of stimulation (resting condition) and in the presence of the mitogens concanavalin A (ConA) and lipopolysaccharide (LPS). B: Stimulation release (relating values in the presence and absence of mitogenic stimulus). *p≤0.05, **p≤0.01, ***p≤0.001, differences between ages. Data is shown in Figure 1.

# TNFα

A

| Resting | NTg | | | | |
|---|---|---|---|---|---|
| 3xTgAD | 2 | 4 | 6 | 12 | 15 |
| 2 | | ** | - | ** | *** |
| 4 | - | | - | * | ** |
| 6 | - | - | | * | ** |
| 12 | *** | *** | * | | - |
| 15 | ** | *** | ** | - | |

| ConA | NTg | | | | |
|---|---|---|---|---|---|
| 3xTgAD | 2 | 4 | 6 | 12 | 15 |
| 2 | | ** | - | *** | ** |
| 4 | - | | * | - | - |
| 6 | - | ** | | *** | *** |
| 12 | * | * | ** | | - |
| 15 | * | * | *** | - | |

| LPS | NTg | | | | |
|---|---|---|---|---|---|
| 3xTgAD | 2 | 4 | 6 | 12 | 15 |
| 2 | | ** | - | * | ** |
| 4 | * | | * | - | - |
| 6 | ** | - | | - | - |
| 12 | - | * | - | | - |
| 15 | ** | ** | ** | ** | |

B

| ConA / Resting | NTg | | | | |
|---|---|---|---|---|---|
| 3xTgAD | 2 | 4 | 6 | 12 | 15 |
| 2 | | - | - | * | * |
| 4 | - | | - | - | - |
| 6 | - | - | | - | - |
| 12 | - | * | - | | - |
| 15 | - | - | - | - | |

| LPS / Resting | NTg | | | | |
|---|---|---|---|---|---|
| 3xTgAD | 2 | 4 | 6 | 12 | 15 |
| 2 | | - | - | - | - |
| 4 | - | | - | - | - |
| 6 | - | - | | - | * |
| 12 | - | - | ** | | - |
| 15 | - | - | - | * | |

C

| ConA / IL-10 | NTg | | | | |
|---|---|---|---|---|---|
| 3xTgAD | 2 | 4 | 6 | 12 | 15 |
| 2 | | *** | *** | *** | * |
| 4 | *** | | *** | - | - |
| 6 | ** | - | | - | - |
| 12 | *** | - | - | | - |
| 15 | ** | - | - | * | |

| LPS / IL-10 | NTg | | | | |
|---|---|---|---|---|---|
| 3xTgAD | 2 | 4 | 6 | 12 | 15 |
| 2 | | * | * | * | - |
| 4 | *** | | - | - | - |
| 6 | *** | - | | - | - |
| 12 | *** | - | - | | - |
| 15 | *** | * | * | * | |

**Figure S2.** Statistical differences of the age-dependent changes in the release of the pro-inflammatory cytokine tumor necrosis factor-alpha (TNF*α*) by peritoneal leukocytes from non-transgenic (NTg) and triple transgenic (3xTg) mice for Alzheimer's disease (3xTgAD). A: In the absence of stimulation (resting condition) and in the presence of the mitogens concanavalin A (ConA) and lipopolysaccharide (LPS). B: Stimulation release (relating values in the presence and absence of mitogenic stimulus). C: TNF*α*/IL-10 ratio. *p≤0.05, **p≤0.01, ***p≤0.001, differences between ages. Data is shown in Figure 2.

## IL-1β

A

| Resting (3xTgAD \ NTg) | 2 | 4 | 6 | 12 | 15 |
|---|---|---|---|---|---|
| 2 | | * | ** | * | - |
| 4 | * | | *** | * | - |
| 6 | ** | ** | | *** | *** |
| 12 | * | * | * | | - |
| 15 | * | * | ** | - | |

| ConA (3xTgAD \ NTg) | 2 | 4 | 6 | 12 | 15 |
|---|---|---|---|---|---|
| 2 | | - | * | - | - |
| 4 | - | | - | - | - |
| 6 | * | *** | | ** | ** |
| 12 | * | ** | ** | | - |
| 15 | - | ** | *** | ** | |

| LPS (3xTgAD \ NTg) | 2 | 4 | 6 | 12 | 15 |
|---|---|---|---|---|---|
| 2 | | * | *** | - | ** |
| 4 | - | | *** | - | * |
| 6 | *** | *** | | *** | *** |
| 12 | * | * | * | | * |
| 15 | - | - | *** | - | |

B

| ConA / Resting (3xTgAD \ NTg) | 2 | 4 | 6 | 12 | 15 |
|---|---|---|---|---|---|
| 2 | | - | - | - | * |
| 4 | - | | - | - | - |
| 6 | - | - | | - | - |
| 12 | - | - | *** | | - |
| 15 | * | - | - | - | |

| LPS / Resting (3xTgAD \ NTg) | 2 | 4 | 6 | 12 | 15 |
|---|---|---|---|---|---|
| 2 | | - | - | - | - |
| 4 | - | | - | - | * |
| 6 | - | - | | - | - |
| 12 | - | - | *** | | - |
| 15 | - | - | - | - | |

C

| ConA / IL-10 (3xTgAD \ NTg) | 2 | 4 | 6 | 12 | 15 |
|---|---|---|---|---|---|
| 2 | | ** | *** | * | - |
| 4 | *** | | *** | ** | *** |
| 6 | - | *** | | *** | *** |
| 12 | *** | ** | *** | | - |
| 15 | *** | - | *** | ** | |

| LPS / IL-10 (3xTgAD \ NTg) | 2 | 4 | 6 | 12 | 15 |
|---|---|---|---|---|---|
| 2 | | - | - | - | * |
| 4 | - | | ** | - | *** |
| 6 | ** | ** | | * | ** |
| 12 | ** | ** | ** | | - |
| 15 | - | - | ** | ** | |

**Figure S3.** Statistical differences of the age-dependent changes in the release of the pro-inflammatory cytokine interleukin one beta (IL-1β) by peritoneal leukocytes from non-transgenic (NTg) and triple transgenic (3xTg) mice for Alzheimer's disease (3xTgAD). A: In the absence of stimulation (resting condition) and in the presence of the mitogens concanavalin A (ConA) and lipopolysaccharide (LPS). B: Stimulation release (relating values in the presence and absence of mitogenic stimulus). C: IL-1β/IL-10 ratio. *p≤0.05, **p≤0.01, ***p≤0.001, differences between ages. Data is shown in Figure 3.

## IL-6

A

| Resting (3xTgAD \ NTg) | 2 | 4 | 6 | 12 | 15 |
|---|---|---|---|---|---|
| 2 | | - | - | *** | * |
| 4 | - | | - | *** | *** |
| 6 | * | - | | *** | *** |
| 12 | *** | *** | *** | | *** |
| 15 | *** | *** | *** | *** | |

| ConA (3xTgAD \ NTg) | 2 | 4 | 6 | 12 | 15 |
|---|---|---|---|---|---|
| 2 | | - | - | - | - |
| 4 | - | | - | * | - |
| 6 | - | - | | * | - |
| 12 | * | * | * | | - |
| 15 | * | ** | ** | - | |

| LPS (3xTgAD \ NTg) | 2 | 4 | 6 | 12 | 15 |
|---|---|---|---|---|---|
| 2 | | - | - | - | - |
| 4 | - | | - | - | - |
| 6 | - | - | | * | * |
| 12 | - | * | ** | | - |
| 15 | - | ** | ** | - | |

B

| ConA / Resting (3xTgAD \ NTg) | 2 | 4 | 6 | 12 | 15 |
|---|---|---|---|---|---|
| 2 | | - | - | - | - |
| 4 | - | | - | - | - |
| 6 | - | - | | - | * |
| 12 | - | - | - | | - |
| 15 | - | - | - | - | |

| LPS / Resting (3xTgAD \ NTg) | 2 | 4 | 6 | 12 | 15 |
|---|---|---|---|---|---|
| 2 | | - | - | - | - |
| 4 | - | | - | - | - |
| 6 | - | - | | - | - |
| 12 | - | - | - | | - |
| 15 | - | - | - | - | |

C

| ConA / IL-10 (3xTgAD \ NTg) | 2 | 4 | 6 | 12 | 15 |
|---|---|---|---|---|---|
| 2 | | *** | *** | * | - |
| 4 | *** | | * | * | * |
| 6 | ** | ** | | *** | ** |
| 12 | ** | * | ** | | - |
| 15 | * | * | ** | - | |

| LPS / IL-10 (3xTgAD \ NTg) | 2 | 4 | 6 | 12 | 15 |
|---|---|---|---|---|---|
| 2 | | * | ** | - | - |
| 4 | * | | * | - | - |
| 6 | * | * | | - | - |
| 12 | ** | - | * | | - |
| 15 | ** | - | - | - | |

**Figure S4.** Statistical differences of the age-dependent changes in the release of the pro-inflammatory cytokine interleukin six (IL-6) by peritoneal leukocytes from non-transgenic (NTg) and triple transgenic (3xTg) mice for Alzheimer's disease (3xTgAD). A: In the absence of stimulation (resting condition) and in the presence of the mitogens concanavalin A (ConA) and lipopolysaccharide (LPS). B: Stimulation release (relating values in the presence and absence of mitogenic stimulus). C: IL-6/IL-10 ratio. *p≤0.05, **p≤0.01, ***p≤0.001, differences between ages. Data is shown in Figure 4.

## INFγ

A

| Resting | NTg | | | | |
|---|---|---|---|---|---|
| 3xTgAD | 2 | 4 | 6 | 12 | 15 |
| 2 | | * | - | - | - |
| 4 | - | | - | * | - |
| 6 | - | * | | * | * |
| 12 | - | * | - | | * |
| 15 | - | - | - | ** | |

| ConA | NTg | | | | |
|---|---|---|---|---|---|
| 3xTgAD | 2 | 4 | 6 | 12 | 15 |
| 2 | | *** | * | * | - |
| 4 | - | | - | * | - |
| 6 | - | - | | - | - |
| 12 | - | - | - | | ** |
| 15 | - | - | - | - | |

| LPS | NTg | | | | |
|---|---|---|---|---|---|
| 3xTgAD | 2 | 4 | 6 | 12 | 15 |
| 2 | | - | ** | - | * |
| 4 | - | | *** | - | * |
| 6 | - | - | | - | * |
| 12 | - | - | - | | - |
| 15 | - | - | - | - | |

B

| ConA / Resting | NTg | | | | |
|---|---|---|---|---|---|
| 3xTgAD | 2 | 4 | 6 | 12 | 15 |
| 2 | | - | - | *** | - |
| 4 | - | | - | - | - |
| 6 | - | - | | - | * |
| 12 | - | - | * | | - |
| 15 | - | - | - | - | |

| LPS / Resting | NTg | | | | |
|---|---|---|---|---|---|
| 3xTgAD | 2 | 4 | 6 | 12 | 15 |
| 2 | | * | - | - | - |
| 4 | *** | | - | - | - |
| 6 | *** | *** | | - | * |
| 12 | *** | - | - | | - |
| 15 | *** | ** | - | - | |

C

| ConA / IL-10 | NTg | | | | |
|---|---|---|---|---|---|
| 3xTgAD | 2 | 4 | 6 | 12 | 15 |
| 2 | | - | - | - | - |
| 4 | ** | | * | * | - |
| 6 | ** | ** | | *** | * |
| 12 | ** | ** | ** | | * |
| 15 | ** | *** | *** | - | |

| LPS / IL-10 | NTg | | | | |
|---|---|---|---|---|---|
| 3xTgAD | 2 | 4 | 6 | 12 | 15 |
| 2 | | * | ** | - | * |
| 4 | * | | *** | * | ** |
| 6 | - | - | | - | - |
| 12 | - | ** | ** | | - |
| 15 | - | * | - | - | |

**Figure S5.** Statistical differences of the age-dependent changes in the release of the pro-inflammatory and regulatory cytokine interferon-gamma (INFγ) by peritoneal leukocytes from non-transgenic (NTg) and triple transgenic (3xTg) mice for Alzheimer's disease (3xTgAD). A: In the absence of stimulation (resting condition) and in the presence of the mitogens concanavalin A (ConA) and lipopolysaccharide (LPS). B: Stimulation release (relating values in the presence and absence of mitogenic stimulus). C: INFγ/IL-10 ratio. *p≤0.05, **p≤0.01, ***p≤0.001, differences between ages. Data is shown in Figure 5.

## IL-17

A

| Resting | NTg | | | | |
|---|---|---|---|---|---|
| 3xTgAD | 2 | 4 | 6 | 12 | 15 |
| 2 | | - | * | ** | - |
| 4 | * | | ** | ** | * |
| 6 | * | * | | ** | ** |
| 12 | * | - | * | | - |
| 15 | * | - | * | * | |

| ConA | NTg | | | | |
|---|---|---|---|---|---|
| 3xTgAD | 2 | 4 | 6 | 12 | 15 |
| 2 | | * | *** | - | * |
| 4 | - | | *** | * | * |
| 6 | - | ** | | * | ** |
| 12 | - | * | - | | - |
| 15 | - | - | * | - | |

| LPS | NTg | | | | |
|---|---|---|---|---|---|
| 3xTgAD | 2 | 4 | 6 | 12 | 15 |
| 2 | | - | * | - | ** |
| 4 | *** | | * | - | ** |
| 6 | - | * | | - | - |
| 12 | *** | ** | * | | - |
| 15 | - | * | * | * | |

B

| ConA / Resting | NTg | | | | |
|---|---|---|---|---|---|
| 3xTgAD | 2 | 4 | 6 | 12 | 15 |
| 2 | | - | - | - | * |
| 4 | - | | * | * | - |
| 6 | - | - | | - | - |
| 12 | - | - | - | | - |
| 15 | - | - | - | - | |

| LPS / Resting | NTg | | | | |
|---|---|---|---|---|---|
| 3xTgAD | 2 | 4 | 6 | 12 | 15 |
| 2 | | - | - | - | - |
| 4 | - | | - | - | *** |
| 6 | - | - | | - | - |
| 12 | - | - | - | | - |
| 15 | - | - | - | - | |

C

| ConA / IL-10 | NTg | | | | |
|---|---|---|---|---|---|
| 3xTgAD | 2 | 4 | 6 | 12 | 15 |
| 2 | | - | ** | * | * |
| 4 | ** | | *** | *** | *** |
| 6 | * | * | | - | * |
| 12 | * | *** | * | | - |
| 15 | * | ** | - | ** | |

| LPS / IL-10 | NTg | | | | |
|---|---|---|---|---|---|
| 3xTgAD | 2 | 4 | 6 | 12 | 15 |
| 2 | | - | * | - | ** |
| 4 | * | | * | * | ** |
| 6 | - | - | | - | - |
| 12 | ** | *** | *** | | - |
| 15 | - | ** | *** | *** | |

**Figure S6.** Statistical differences of the age-dependent changes in the release of the pro-inflammatory and regulatory cytokine interleukin seventeen (IL-17) by peritoneal leukocytes from non-transgenic (NTg) and triple transgenic (3xTg) mice for Alzheimer's disease (3xTgAD). A: In the absence of stimulation (resting condition) and in the presence of the mitogens concanavalin A (ConA) and lipopolysaccharide (LPS). B: Stimulation release (relating values in the presence and absence of mitogenic stimulus). C: IL-17/IL-10 ratio. *p≤0.05, **p≤0.01, ***p≤0.001, differences between ages. Data is shown in Figure 6.

# IL-2

A

| Resting | NTg 2 | NTg 4 | NTg 6 | NTg 12 | NTg 15 |
|---|---|---|---|---|---|
| 3xTgAD 2 | | - | - | - | - |
| 3xTgAD 4 | - | | - | - | - |
| 3xTgAD 6 | - | - | | - | - |
| 3xTgAD 12 | - | - | - | | - |
| 3xTgAD 15 | - | - | - | - | |

| ConA | NTg 2 | NTg 4 | NTg 6 | NTg 12 | NTg 15 |
|---|---|---|---|---|---|
| 3xTgAD 2 | | ** | ** | ** | * |
| 3xTgAD 4 | * | | - | * | - |
| 3xTgAD 6 | - | - | | ** | - |
| 3xTgAD 12 | ** | ** | *** | | - |
| 3xTgAD 15 | * | * | * | - | |

| LPS | NTg 2 | NTg 4 | NTg 6 | NTg 12 | NTg 15 |
|---|---|---|---|---|---|
| 3xTgAD 2 | | * | - | - | - |
| 3xTgAD 4 | - | | - | - | - |
| 3xTgAD 6 | - | ** | | * | * |
| 3xTgAD 12 | ** | - | * | | - |
| 3xTgAD 15 | - | - | - | - | |

B

| ConA / Resting | NTg 2 | NTg 4 | NTg 6 | NTg 12 | NTg 15 |
|---|---|---|---|---|---|
| 3xTgAD 2 | | *** | *** | * | - |
| 3xTgAD 4 | * | | - | * | - |
| 3xTgAD 6 | - | * | | * | - |
| 3xTgAD 12 | * | * | ** | | - |
| 3xTgAD 15 | - | - | * | - | |

| LPS / Resting | NTg 2 | NTg 4 | NTg 6 | NTg 12 | NTg 15 |
|---|---|---|---|---|---|
| 3xTgAD 2 | | * | - | - | - |
| 3xTgAD 4 | - | | * | - | - |
| 3xTgAD 6 | - | - | | - | * |
| 3xTgAD 12 | ** | - | * | | - |
| 3xTgAD 15 | - | - | - | - | |

**Figure S7.** Statistical differences of the age-dependent changes in the release of the regulatory cytokine interleukin two (IL-2) by peritoneal leukocytes from non-transgenic (NTg) and triple transgenic (3xTg) mice for Alzheimer's disease (3xTgAD). A: In the absence of stimulation (resting condition) and in the presence of the mitogens concanavalin A (ConA) and lipopolysaccharide (LPS). B: Stimulation release (relating values in the presence and absence of mitogenic stimulus). *p≤0.05, **p≤0.01, ***p≤0.001, differences between ages. Data is shown in Figure 1.
